# Reactive Oxygen Species Evoked by Potassium Deprivation and Staurosporine Inactivate Akt and Induce the Expression of TXNIP in Cerebellar Granule Neurons

**DOI:** 10.1155/2017/8930406

**Published:** 2017-03-06

**Authors:** Marco Antonio Zaragoza-Campillo, Julio Morán

**Affiliations:** División de Neurociencias, Instituto de Fisiología Celular, Universidad Nacional Autónoma de México, Ciudad de México, Mexico

## Abstract

The reactive oxygen species (ROS) play a critical role in neuronal apoptosis; however, the mechanisms are not well understood. It has been shown that thioredoxin-interacting protein (TXNIP) overexpression renders cells more susceptible to oxidative stress and promotes apoptosis and that the activation of PI3K/Akt pathway leads to a downregulation of TXNIP. Here, we evaluated the role of ROS in the regulation of Akt activity and the subsequent regulation of the TXNIP expression in a model of apoptotic death of cerebellar granule neurons (CGN). We observed that two apoptotic conditions that generate ROS at short times led to an increase in the expression of TXNIP in a time-dependent manner; antioxidants significantly reduced this expression. Also, H_2_O_2_ caused an increase in TXNIP expression. Moreover, apoptotic conditions induced inactivation of Akt in a time-dependent manner similar to TXNIP expression and H_2_O_2_ treatment led to Akt inactivation. Besides, the pharmacological inhibition of Akt increases TXNIP expression and induces CGN cell death. Together, these results suggest that ROS promote neuronal apoptosis through the Akt-TXNIP signaling pathway, supporting the idea that the PI3K/Akt pathway regulates the TXNIP expression. This study highlights the potential importance of this mechanism in neuronal death.

## 1. Introduction

Several studies have demonstrated that reactive oxygen species (ROS) regulate diverse physiological processes through a direct oxidation of specific proteins. This oxidative process leads to a modification in the functionality of the target proteins. Several proteins have been reported to be regulated by its redox state; these include channels, receptors, and structural and signaling proteins. ROS act mainly through the oxidation of specific amino acid residues, such as cysteines [1] to control protein function. For example, several studies have demonstrated that hydrogen peroxide (H_2_O_2_) regulates some physiological processes by modulating the activity of several protein kinases and protein phosphatases through the oxidation of cysteines [2–5].

In addition, some proteins regulate the redox state of other proteins that are involved in the control of the oxidative levels in the cell, as well as the activation/inactivation of multiple signaling pathways. That is the case of the thioredoxin-interacting protein (TXNIP), also known as VDUP1 or TBP-2. This protein binds to and negatively regulates thioredoxin 1 (Trx1) that controls the cellular redox state [6–10] and protects cells against deleterious actions of ROS, including cell death [11]. Trx1 also modulates the activation of ASK1, a member of the mitogen-activated protein kinase pathway, which is involved in apoptotic death and responds to oxidative stress [12]. TXNIP expression is ubiquitous and is induced by a variety of stress conditions, including UV light, *γ*-rays, heat shock, and H_2_O_2_ [7, 13]. Cells overexpressing TXNIP are more susceptible to oxidative stress and apoptosis [14–17]. Recently, it was shown that TXNIP is essential for glucotoxicity-induced apoptosis of beta cell mediated by the intrinsic mitochondrial apoptotic pathway [18].

It has been reported that the PI3K/Akt signaling pathway regulates the expression of TXNIP [19, 20]. In cancer cells, it has been suggested that the activation of PI3K/Akt leads to a downregulation of TXNIP at the transcriptional level. Conversely, inhibition of the activated PI3K/Akt pathway in lung cancer cells increases TXNIP expression [19]. Similar results were observed in different cell lines, suggesting that the downregulation of TXNIP by growth factors is a ubiquitous phenomenon and that the constitutive activation of PI3K/Akt/mTOR leads to TXNIP downregulation [19]. These findings are consistent with a previous study showing serum-induced TXNIP downregulation in fibroblasts and T-lymphocytes [21].

Akt is a protein kinase involved in different physiological processes such as cell proliferation, migration, and apoptosis. Akt regulates some members of the Bcl-2 super family and indirectly controls apoptosis through the transcriptional factors that mediate apoptotic events [22]. Several studies have shown that Akt activity can be directly modified by ROS. It has been reported that the exposure of cardiac cells to H_2_O_2_ induces the formation of an intramolecular disulfide bond between Cys297 and Cys311 of the Akt kinase domain, which leads to its dephosphorylation and proteasomal degradation [23]. Conversely, under cytotoxic stress, the prevention of oxidation of these cysteine residues reduces both its dephosphorylation and proteasomal degradation, which translates into an enhanced cell survival [24]. In addition, it has been found that MPTP, a neurotoxin that selectively damages dopaminergic neurons, induces an oxidation of specific Akt cysteines, which increases its association with PP2A, reducing its level of phosphorylation. The presence of antioxidants fully reverses these effects and reduces MPTP toxicity [25]. These studies strongly suggest that Akt is a redox-sensitive protein, a condition that may modulate its involvement in the cell survival.

Neurons are susceptible to oxidative damage because of their high levels of ROS production and relatively low levels of antioxidant enzymes [26]. Since TXNIP plays a significant role in the cellular redox state, we investigated the possibility that the apoptotic conditions that induce ROS generation could regulate the expression of TXNIP, as well as the activity of Akt in neurons. We evaluated this possibility by using a model of apoptotic death of cultured cerebellar granule neurons (CGN) induced by potassium deprivation (K5) and staurosporine (Sts). Under these conditions, we provide evidence that ROS generated by proapoptotic stimuli led to an upregulation of TXNIP, as well as an inactivation of Akt kinase. The inactivation of Akt induced TXNIP expression. These results support the idea that both TXNIP and Akt are proteins sensitive to the redox state depending on apoptotic conditions in cerebellar granule neurons and raise the possibility that Akt is involved in the regulation of TXNIP expression under these conditions.

## 2. Materials and Methods

### 2.1. Materials

Fetal calf serum, penicillin/streptomycin, and basal Eagle's medium were purchased from GIBCO, Invitrogen (Carlsbad, CA, USA). Dihydroethidium (DHEt) was purchased from Molecular Probes, Invitrogen (Carlsbad, CA, USA). Poly-l-lysine (mol. wt. > 300,000), trypsin, trypsin inhibitor, DNAse, cytosine arabinoside, DMSO, staurosporine, 4-(2-aminoethyl) benzenesulfonyl fluoride hydrochloride (AEBSF), LY 294002, and reagents for polyacrylamide gel electrophoresis (PAGE) were acquired from Sigma (St. Louis, MO, USA). Protease inhibitor cocktail tablets (Complete) were purchased from Roche (Mannheim, Germany) and phosphatase inhibitor mini tablets were obtained from Thermo Scientific (Rockford, USA). ProSieve Quadcolor Protein Marker was purchased from Lonza (Rockland, Maine, USA). Polyvinylidene fluoride (PVDF) membranes and Immobilon Western HRP substrate were acquired from Millipore (Concord Road, Billerica, MA, USA). CDP-Star Reagent for alkaline phosphatase-based chemiluminescent detection assays was purchased from New England Biolabs and BioMax Light Film was purchased from Kodak (Rochester, NY, USA). Ebselen and EUK-134 were from Cayman Chemical (Ann Arbor, MI, USA). Antibodies against Akt and p-Akt (Ser473) were from Cell Signaling Technology (Danvers, MA, USA). Antibody against TXNIP was purchased from Novus Biologicals. Antibody against glyceraldehyde 3-phosphate dehydrogenase (GAPDH) was from Millipore (Bedford, MA, USA). Peroxidase-conjugated anti-mouse was purchased from Jackson ImmunoResearch (West Grove, PA, USA), and alkaline phosphatase-conjugated anti-mouse and anti-rabbit were purchased from Sigma (St. Louis, MO, USA).

### 2.2. Experimental Model

Cultured isolated cerebellar granule neurons (CGN) are widely employed as a model of apoptosis. Notably, rat CGN die when maintained in vitro for more than 5 DIV with physiological [K^+^] (3.5–5 mM) under no depolarizing conditions (K5). To avoid this condition, CGN are chronically cultivated in high [K^+^] (25 mM); under sustained depolarization (K25) and by switching to K5 or vice versa it is possible to alter cell survival and evaluate gene expression, neurochemical maturation, and physiological properties of these cells [27].

On the other hand, staurosporine (Sts) has been shown to induce apoptosis in wide variety of cell types. It has been believed that the proteins required to execute staurosporine-induced programmed cell death are constitutively present in all nucleated mammalian cells [28]. Staurosporine induces apoptotic cell death at 0.2–1 *µ*M [29–32] and has been recognized as a useful model to investigate the mechanism of apoptosis in mammalian cells [31–33]. Based on this, in the present study we employed both conditions (potassium deprivation and staurosporine) as inducers of apoptotic death.

### 2.3. Cell Cultures

All animals used for the experimentation described in the present study were treated in accordance with the accepted standards of animal care and with the procedures approved by the local Committee of Research and Ethics of the Instituto de Fisiología Celular, Universidad Nacional Autónoma de México. The protocol used followed the Guidelines for the Care and Use of Mammals in Neuroscience as well as guidelines released by the Mexican Institutes of Health Research and the National Institutes of Health guide for the care and use of Laboratory animals. All efforts were made to minimize animal suffering and to reduce the number of animals used.

Cerebellar granule neurons (CGN) cultures were prepared as previously described [34]. Briefly, cell suspensions dissociated from 8-day-old Wistar rat cerebellum were plated at a density of 265 × 10^3^ cells/cm^2^ in plastic dishes coated previously with poly-l-lysine (5 *μ*g/mL). The culture medium contained basal Eagle's medium supplemented with 10% (v/v) heat-inactivated fetal calf serum, 2 mM glutamine, 25 mM KCl, 50 *μ*g/mL streptomycin, and 50 U/mL penicillin. Cytosine arabinoside (10 *μ*M) was added 24 h after seeding to prevent the proliferation of nonneuronal cells. The cultures were kept at 37°C in an atmosphere of CO_2_ (5%) and saturated air with water vapor (95%). This medium is referred to as K25. At the end of the preparation, CGN cultures contained approximately 95% neurons. Cultures were maintained 7 days in the depolarizing medium (K25) and subsequently apoptotic death of CGN was induced by two different stimuli: (a) the neurons were transferred to a serum-free medium containing 5 mM KCl (referred as K5) or (b) by the administration of 0.5 *µ*M staurosporine (Sts) for varying times (0.25–5.5 h).

### 2.4. Determination of ROS

It is known that dihydroethidium (DHEt) diffuses into cells and is oxidized by reactive oxygen species in the cytosol producing ethidium and 2-hydroxyethidium that binds to the DNA and emits bright red fluorescence. Thus, fluorescence intensity is an indirect parameter of ROS levels. CGN were maintained in a K25 medium during 7 days in vitro (DIV) and after this time the CGN were transferred to a medium with K5 or treated with Sts and maintained in these conditions from 0.5 h to 5.5 h. After treatment, the cells were incubated with 3.2 *µ*M DHEt for 20 minutes at 37°C. Then, cells were washed with PBS and observed in a fluorescence microscope with a rhodamine filter with a wavelength of 488–515 nm. Cells were photographed and fluorescence intensity was measured with the Image J Program. ROS production by xanthine/xanthine oxidase was used as positive control. For that, 7-8 DIV cultures were incubated with 10 *µ*M xanthine (X) for 1 h and then xanthine oxidase (XO) (45 mU/mL) was added to the medium and ROS generation was evaluated after 2 h [35].

### 2.5. Cell Viability

Cell viability was determined by the accumulation of calcein-AM and exclusion of propidium iodide (IP) to stain live and dead cells, respectively. The viable cells contain endogenous esterases and are identified by their ability to convert calcein-AM to calcein, a green fluorescent product. The IP crosses the plasma membrane of damaged cells; it binds to DNA and double-stranded RNA by intercalation and emits intense red fluorescence signal. This dye shows weak fluorescence when it is not bound and in aqueous solution. Cells were incubated with calcein-AM (1 *µ*M) and IP (40 *µ*M) for 15 min at 37°C and were observed in a fluorescence microscope and stained cells were counted.

We additionally evaluated cell viability by the MTT (3-(4, 5-dimethylthiazol-2-yl)-2, 5-diphenyltetrazolium bromide) reduction technique, which is based on the ability of mitochondrial succinate dehydrogenase to transform MTT to formazan blue. Since only living cells carry out this reaction, the amount of formazan produced is directly proportional to the number of viable cells present in the culture. The cells were incubated with MTT (100 *µ*M) during 15 min at 37°C, cultures were washed with saline solution and formazan blue was extracted with DMSO (dimethyl sulphoxide, 100%), and the amount was calculated by reading in the spectrophotometer at 570 nm.

### 2.6. Immunoblotting

CGN were cultured in a K25 medium for 7 DIV and then switched to a K5 medium or treated with staurosporine from 15 min to 5.5 h. Some cultures were treated with hydrogen peroxide, antioxidants, or NADPH-oxidase inhibitors. Cells were washed twice in ice-cold PBS and homogenized in lysis buffer (25 mM Trizma, 50 mM NaCl, 2% Igepal, 0.2% SDS, and complete protease inhibitors, pH 7.4). Homogenates were sonicated and centrifuged at 4,500 rpm for 5 min and the supernatants were recovered. The protein concentration of cellular homogenates was determined by the Bradford method. Homogenates (60 *µ*g protein per lane) were subjected to 15% SDS-PAGE and transferred to PVDF membranes at 100 V for 1.5 h. The membranes were blocked with Tris-buffered saline (TBS)/Tween 20 (TTBS) buffer (100 mM Tris–HCl, 150 mM NaCl and 0.1% Tween, pH 7.4) containing 5% or 2.5% nonfat dry milk at 4°C overnight and incubated overnight at 4°C with the primary antibodies. After further washing, the blots were incubated with peroxidase-conjugated anti-mouse (1 : 10,000) or alkaline phosphatase-conjugated anti-rabbit (1 : 20,000) and anti-mouse (1 : 30,000) for 1 h at room temperature. Bands were visualized using chemiluminescence according to the manufacturer's recommendations and exposed to Kodak BioMax-Light Film.

### 2.7. Antibodies

The following antibodies were used: anti-GAPDH (#MAB374), anti-pAkt S473 (D9E) (#4060S), anti-Akt pan (C67E7) (#4691L), and anti-TXNIP (JY2) (#NBP1-S4578). Antibodies used were as follows: 1 : 1000 rabbit anti-pAkt, 1 : 1000 rabbit anti-Akt, 1 : 500 mouse anti-TXNIP, and 1 : 3000 mouse anti-GAPDH.

### 2.8. Statistical Analysis

**Figure 1:**
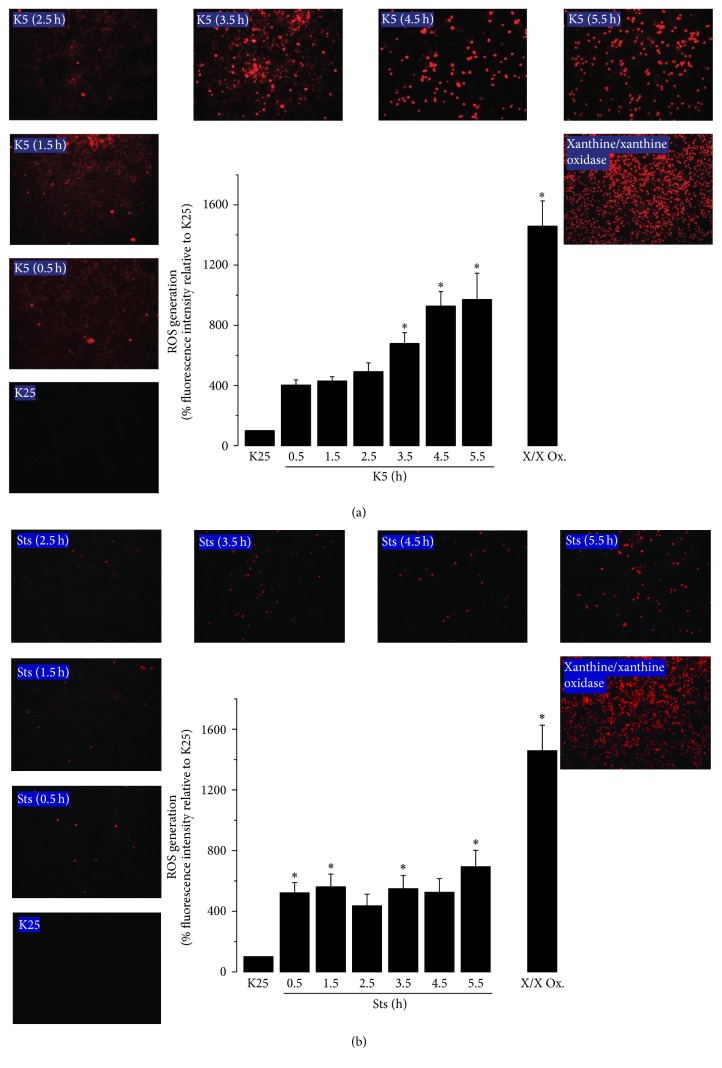
K5 and Sts induce the generation of reactive oxygen species. Primary cultures of rat cerebellar granule neurons (CGN) were cultured with K25 as described in Materials and Methods. After 7 days in vitro (DIV), cells were transferred to K5 medium or were treated with Sts (0.5 *µ*M) from 0.5 h to 5.5 h. ROS levels are expressed as the percentage of mean fluorescence intensity of ethidium cation (product of the dihydroethidium oxidation) with respect to K25. The xanthine/xanthine oxidase treatment was used as a positive control. Data are means ± SE of 5 independent experiments. ^*∗*^*p* < 0.05, significantly different from K25. X/X Ox: xanthine/xanthine oxidase system.

Statistical analysis was done by using SigmaPlot 11.0 software. Data are presented as mean ± SE and statistical significance of the results was determined by one-way analysis of variance (ANOVA) followed by Bonferroni's test. *p* values less than 0.05 were considered statistically significant, indicating the number of experiments. Statistical significance of data from Figure 1 was determined by a nonparametric analysis followed by Dunnett's post hoc test.

## 3. Results

### 3.1. Apoptotic Conditions Induce the Generation of ROS in CGN

In order to elucidate the effect of apoptotic conditions in ROS production in CGN, neuronal cultures maintained in K25 medium for 7 DIV were switched to K5 medium or treated with Sts for 0.5, 1.5, 2.5, 3.5, 4.5, or 5.5 h and ROS levels were measured as detailed in Material and Methods. In accordance with previous results [36], CGN treated with K5 showed an increase in ROS levels from 0.5 h and reached a maximum at 5.5 h (Figure 1(a), Suppl. Fig.  1(A)). Similarly, we found that the maximum level of ROS generation induced by Sts was reached also at 5.5 h after initial treatment; however, the intensity of DHEt positive cells was relatively lower than that observed in K5 condition (Figure 1(b), Suppl. Fig.  1(B)).

Furthermore, we measured the ROS generation in cells treated with xanthine/xanthine oxidase as positive control and we found an increase of 26.20%  ±  4.39 of DHEt positive cells (Figure 1, Suppl. Fig.  1, in Supplementary Material available online at https://doi.org/10.1155/2017/8930406). This approach has been reported to generate superoxide anion; however, it has been shown that exogenously generated superoxide is spontaneously dismutated into hydrogen peroxide in a very short time [37, 38]. Hydrogen peroxide can freely diffuse into the cells and exert its effects, including cell death.

As previously shown [31], K5 induced a decrease in cell viability of 20–25% approximately after 8 h and by 60–70% after 24 h (Supplementary Fig.  2). In the case of staurosporine [31], 50–60% cell death was observed after 24 h (Supplementary Fig.  3). These data indicate that the apoptotic conditions induced by K5 and Sts induce an early ROS production in CGN.

### 3.2. K5 Induces TXNIP Expression in CGN

**Figure 2:**
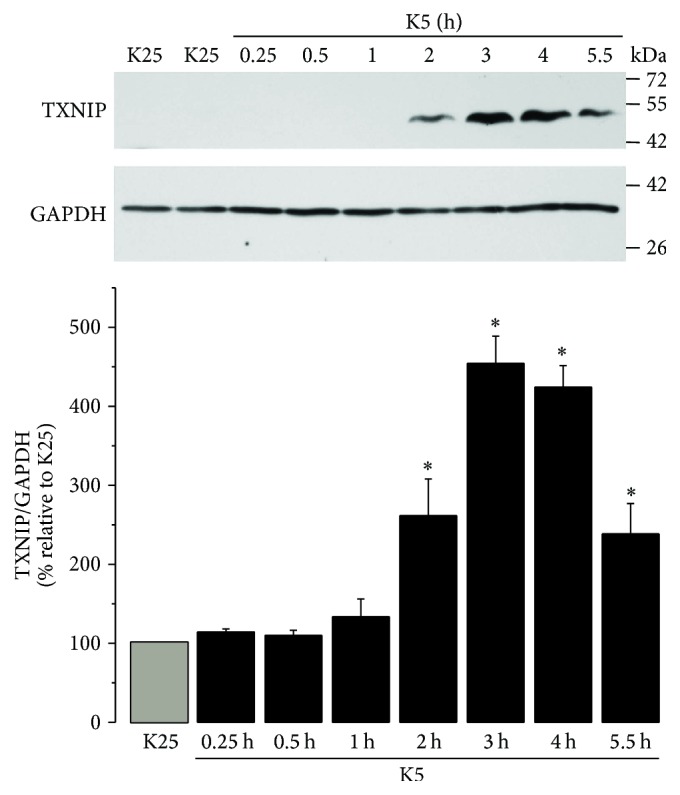
K5 increases the expression of TXNIP. Primary cultures of rat cerebellar granule neurons (CGN) were cultured with K25 as described in Materials and Methods. After 7 DIV cells were treated with K5 during the indicated times and the levels of TXNIP were evaluated as described. Bars indicate the densitometric values of the TXNIP/GAPDH ratio. Results are expressed as the percentage of K25. GAPDH is the load control. TXNIP: 50 kDa, GAPDH: 37 kDa. Data are means ± SE of 4 independent experiments. ^*∗*^*p* < 0.05, significantly different from K25.

The expression pattern of TXNIP was analyzed in cells treated with K5 from 0.25 to 5.5 h. Under these conditions, we observed that the expression of TXNIP started after 2 h of treatment. The TXNIP expression was further increased at 3 h and 4 h, and it decreased after 5.5 h (Figure 2). It is interesting to note that in depolarizing conditions (K25) there was practically no expression of TXNIP (Figure 2).

### 3.3. ROS Inhibitors Prevent TXNIP Expression Induced by K5

**Figure 3:**
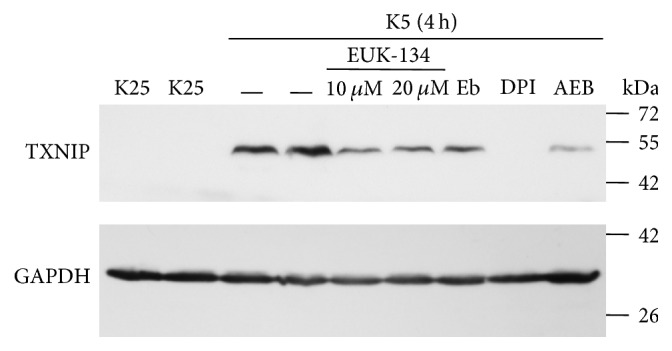
Antioxidants inhibit the expression of TXNIP induced by K5. Primary cultures of rat cerebellar granule neurons (CGN) were cultured with K25 as described in Materials and Methods. After 7 DIV cells were treated with K5 during 4 hours and the levels of TXNIP were evaluated as described. K25 was used as negative control and K5 (4 h) was used as positive control. In K5 (4 h), cells were preincubated with antioxidants EUK-134 (10 and 20 *µ*M) and Ebselen (10 *µ*M), as well as with the NOX inhibitors DPI (520 nm) and AEBSF (50 *µ*M). The immunoblot was performed against TXNIP and GAPDH. GAPDH is the load control. TXNIP: 50 kDa, GAPDH: 37 kDa. Representative blot from 3 independent assays. Eb, Ebselen; AEB, AEBSF.

To elucidate the possible role of ROS in the TXNIP expression induced by K5, we evaluated the effect of the antioxidants EUK-134 and Ebselen, as well as two inhibitors of NOX, DPI, and AEBSF, on TXNIP expression induced by K5. Under these conditions, we observed that 10 and 20 *μ*M EUK-134 and 10 *μ*M Ebselen significantly reduced TXNIP expression induced by K5 at 4 h. Moreover, both NOX inhibitors are more efficient than antioxidants in reducing the TXNIP expression induced by K5, particularly DPI that completely reduced the TXNIP expression (Figure 3). Together, these results suggest that ROS could be mediators in the TXNIP expression induced by potassium deprivation in CGN.

### 3.4. H_2_O_2_ Induces TXNIP Expression in CGN

**Figure 4:**
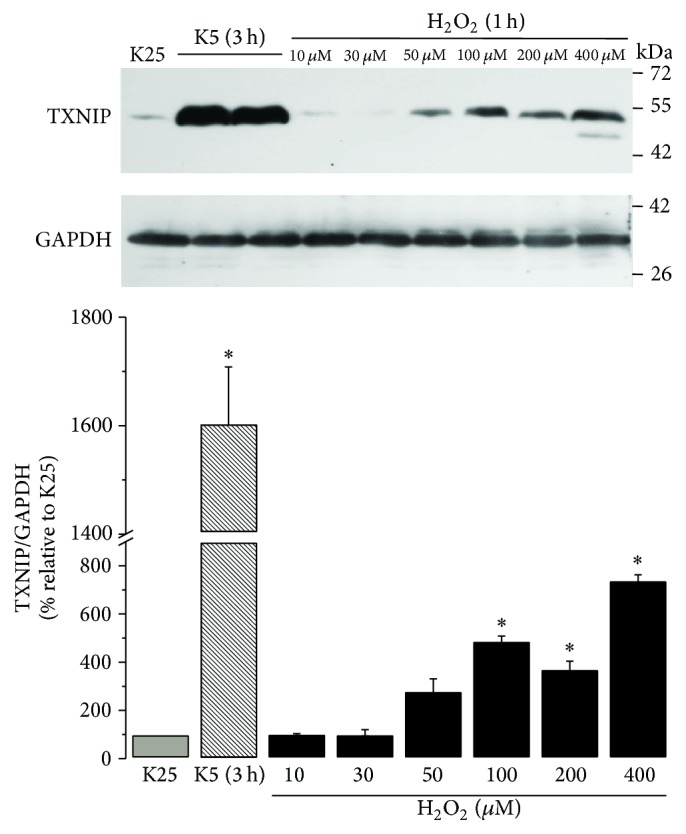
H_2_O_2_ induces the expression of TXNIP. Primary cultures of rat cerebellar granule neurons (CGN) were cultured with K25 as described in Materials and Methods. After 7 DIV, cells were treated with H_2_O_2_ at different concentrations and the levels of TXNIP were evaluated as described. K25 was used as negative control and K5 (3 h) was used as positive control. Bars indicate the densitometric values of the TXNIP/GAPDH ratio. Results are expressed as the percentage of K25. GAPDH is the load control. TXNIP: 50 kDa, GAPDH: 37 kDa. Data are means ± SE of 3 independent experiments. ^*∗*^*p* < 0.05, significantly different from K25.

To evaluate the role of ROS in the TXNIP expression, we analyzed the effect of H_2_O_2_.  Figure 4 shows that TXNIP expression was induced upon H_2_O_2_ treatment under K25 conditions. The effect of H_2_O_2_ depended on the concentration. TXNIP expression increased at 100 *µ*M and reached a maximal expression at the highest concentration tested (400 *µ*M).

### 3.5. TXNIP Expression Induced by Sts Is Mediated by ROS

**Figure 5:**
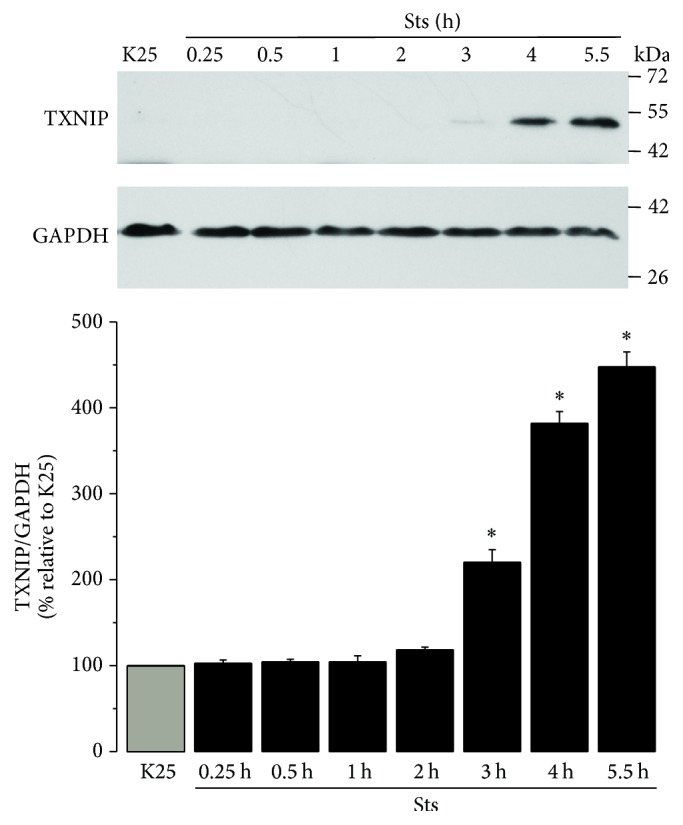
Sts increases the expression of TXNIP. Primary cultures of rat cerebellar granule neurons (CGN) were cultured with K25 as described in Materials and Methods. After 7 DIV cells were treated with Sts (0.5 *µ*M) during the indicated times and the levels of TXNIP were evaluated as described. Bars indicate the densitometric values of the TXNIP/GAPDH ratio. Results are expressed as the percentage of K25. GAPDH is the load control. TXNIP: 50 kDa, GAPDH: 37 kDa. Data are means ± SE of 3 independent experiments. ^*∗*^*p* < 0.05, significantly different from K25.

In order to evaluate the effect of a different apoptotic condition on TXNIP expression, we analyzed the effect of staurosporine (0.5 *µ*M). Under these conditions, we observed that Sts treatment induced an increase in the expression of TXNIP at around 3 h, which continued increasing until 5.5 h (Figure 5). The initial effect of Sts on TXNIP expression is observed later than that observed for the action of K5 (Figure 2).

**Figure 6:**
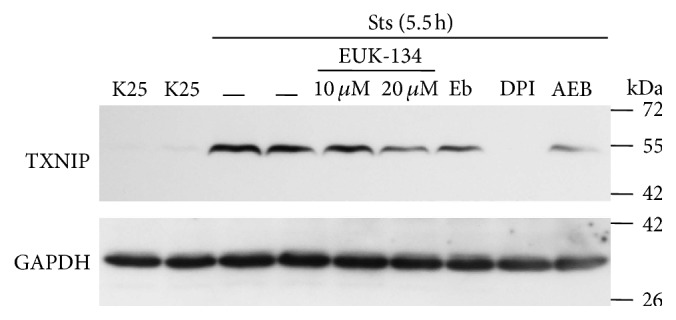
Antioxidants inhibit the expression of TXNIP induced by Sts. Primary cultures of rat cerebellar granule neurons (CGN) were cultured with K25 as described in Materials and Methods. After 7 DIV cells were treated with staurosporine (0.5 *µ*M) during 5.5 hours and the levels of TXNIP were evaluated as described. K25 was used as negative control and Sts (5.5 h) was used as positive control. In Sts (5.5 h), cells were preincubated with antioxidants EUK-134 (10 and 20 *µ*M) and Ebselen (10 *µ*M), as well as with the NOX inhibitors DPI (520 nm) and AEBSF (50 *µ*M). The immunoblot was performed against TXNIP and GAPDH. GAPDH is the load control. TXNIP: 50 kDa, GAPDH: 37 kDa. Representative blot from 3 independent assays. Eb, Ebselen; AEB, AEBSF.

To elucidate the possible role of ROS in the TXNIP expression induced by Sts, we evaluated the effect of the antioxidants EUK-134 and Ebselen, as well as DPI and AEBSF on TXNIP expression induced by Sts. We observed that TXNIP expression induced by Sts at 5.5 h was reduced by EUK-134 (10 and 20 *µ*M), Ebselen (10 *µ*M), and AEBSF (50 *µ*M). Similarly to K5, the expression induced by Sts was also completely inhibited by DPI (520 nM) (Figure 6), suggesting a relevant role of a NADPH-oxidase in the TXNIP transcription induced by Sts.

### 3.6. K5 and Sts Reduce Akt Activation in CGN

**Figure 7:**
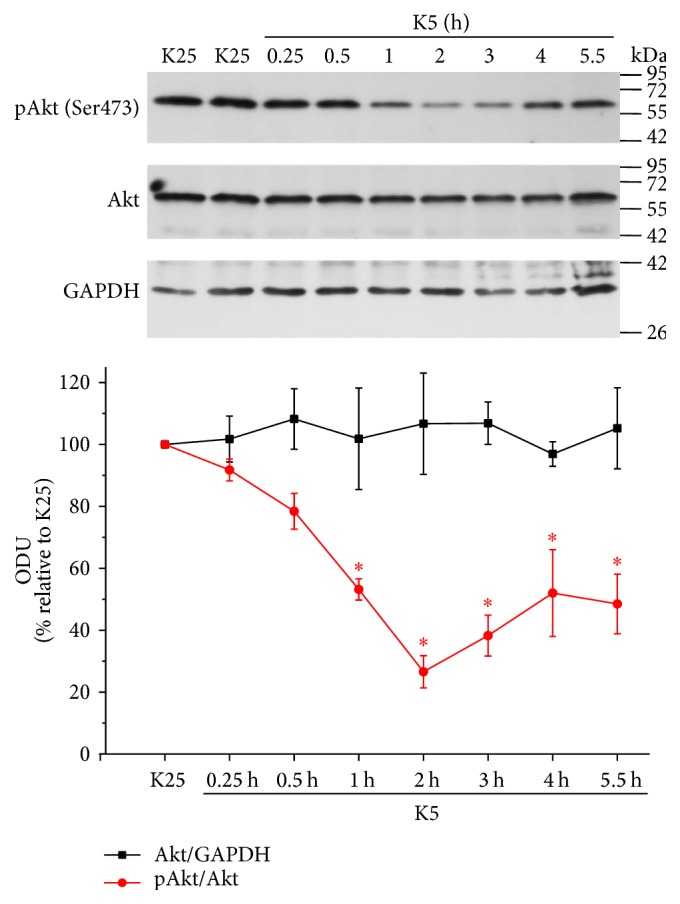
K5 decreases the activation of Akt. Primary cultures of rat cerebellar granule neurons (CGN) were cultured with K25 as described in Materials and Methods. After 7 DIV, cells were treated with K5 during the indicated times and the levels of Akt phosphorylated at the Serine 473 (phosphoserine that monitors the activation status of Akt), total Akt, and GAPDH were evaluated as described. Graph indicates the densitometric values of the Akt/GAPDH ratio and pAkt/Akt ratio. Results are expressed as the percentage of K25. GAPDH is the load control. Akt: 60 kDa, GAPDH: 37 kDa. ODU: Optical Density Units. Values are means ± SE of 3 independent experiments. ^*∗*^*p* < 0.05, significantly different from K25.

It has been reported that a downregulation of PI3K/Akt signaling leads to an increase in the expression of TXNIP [19, 20]. Therefore, in order to explore whether Akt activity is regulated by apoptotic conditions and H_2_O_2_ as it is observed for TXNIP expression, we performed a time course activation of Akt in neurons treated with K5 for 15 min to 5.5 h. The levels of pAkt (Ser473), Akt, and GAPDH were evaluated by Western blot analysis. Under these conditions, we found a significant decrease in phosphorylated Akt (pAkt) levels from one hour of treatment, reaching a maximum of Akt inactivation at 2 and 3 h with respect to K25. Subsequently, a slight recovery in the Akt activation occurred at longest times (Figure 7). Interestingly, these results closely correlate with the temporal pattern of expression of TXNIP induced by K5 (Figure 2).

**Figure 8:**
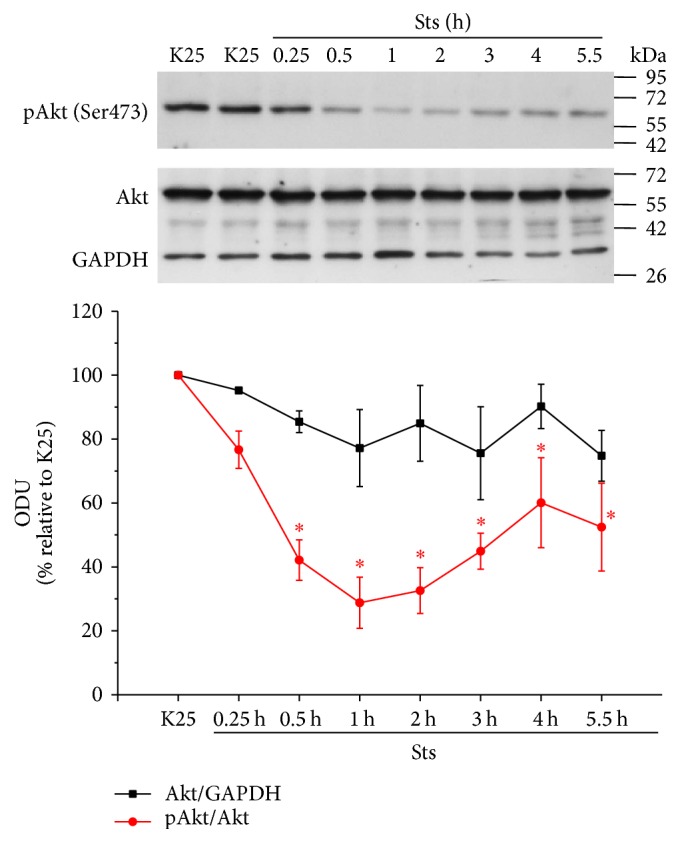
Sts decreases the activation of Akt. Primary cultures of rat cerebellar granule neurons (CGN) were cultured with K25 as described in Materials and Methods. After 7 DIV cells were treated with Sts (0.5 *µ*M) during the indicated times and the levels of Akt phosphorylated at the Serine 473 (Ser473), total Akt, and GAPDH were evaluated as described. Lines indicate the densitometric values of the Akt/GAPDH ratio and pAkt/Akt ratio. Results are expressed as the percentage of K25. GAPDH is the load control. Akt: 60 kDa, GAPDH: 37 kDa. ODU: Optical Density Units. Values are means ± SE of 3 independent experiments. ^*∗*^*p* < 0.05, significantly different from K25.

Similarly, we performed a time course of neurons treated with Sts for 15 min to 5.5 h. Under these conditions, we found that Sts inactivated Akt after 30 min and this effect remained for at least 5.5 h after treatment, showing Akt inactivation at short times in CGN (Figure 8). In addition, we observed a time gap between the Akt inactivation and TXNIP expression (Figure 5); this may be due to temporary differences in the upstream mechanisms that are activated by Sts. The results so far suggest that different apoptotic conditions that induce ROS generation are able to downregulate Akt activation.

### 3.7. H_2_O_2_ Abolishes Akt Activation in CGN

**Figure 9:**
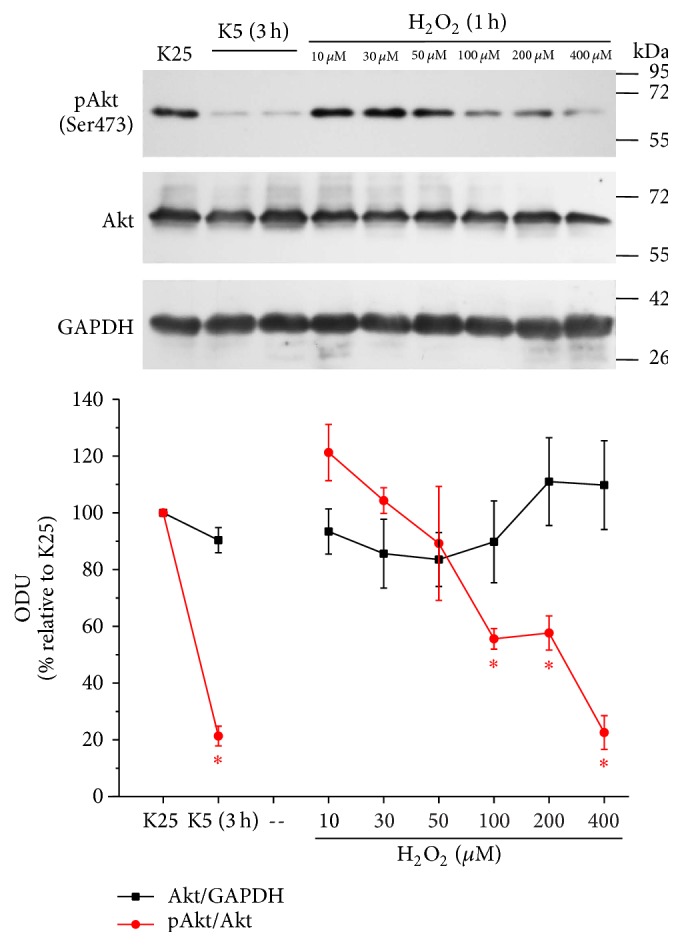
H_2_O_2_ decreases the activity of Akt. Primary cultures of rat cerebellar granule neurons (CGN) were cultured with K25 as described in Materials and Methods. After 7 DIV, cells were treated with H_2_O_2_ at different concentrations and the levels of Akt phosphorylated at the Serine 473 (Ser473), total Akt, and GAPDH were evaluated as described above. K25 was used as negative control and K5 (3 h) was used as positive control. Lines indicate the densitometric values of the Akt/GAPDH ratio and pAkt/Akt ratio. Results are expressed as the percentage of K25. GAPDH is the load control. Akt: 60 kDa, GAPDH: 37 kDa. ODU: Optical Density Units. Values are means ± SE of 3 independent experiments. ^*∗*^*p* < 0.05, significantly different from K25.

We analyzed the effect H_2_O_2_ to elucidate the possible role of ROS in the Akt regulation. Under K25 conditions, we detected that Akt phosphorylation at Ser473 decreased upon H_2_O_2_ treatment. Akt inactivation occurs from 100 *µ*M, showing that inactivation of this kinase is dependent on the concentration of H_2_O_2_. We observed that the Akt inactivation induced by H_2_O_2_ was similar to that observed with K5 (3 h), suggesting that the Akt inactivation induced by K5 is probably mediated by H_2_O_2_ (Figure 9). These data suggest that the regulation of Akt activity is redox sensitive in this model. These results were also similar to the observed effects of H_2_O_2_ on TXNIP expression (Figure 4).

All together, these data strongly suggest that both the Akt activation and the TXNIP expression are regulated by ROS in this model of neuronal death. In order to evaluate this possibility, we measured the levels of TXNIP in cells treated with an inhibitor of Akt activity. To that we used the compound LY 294002, which is a strong inhibitor of PI3K-Akt signaling pathway [39] that has been shown to reduce Akt phosphorylation in several models [40–44]. We first measured the effect of LY 294002 on cell viability in CGN and we found that the inhibition of Akt by LY 294002 induced a significant increase in cell death after 24 h of treatment. Cell viability was measured by the calcein-propidium iodide technique (Supplementary Fig.  4).

**Figure 10:**
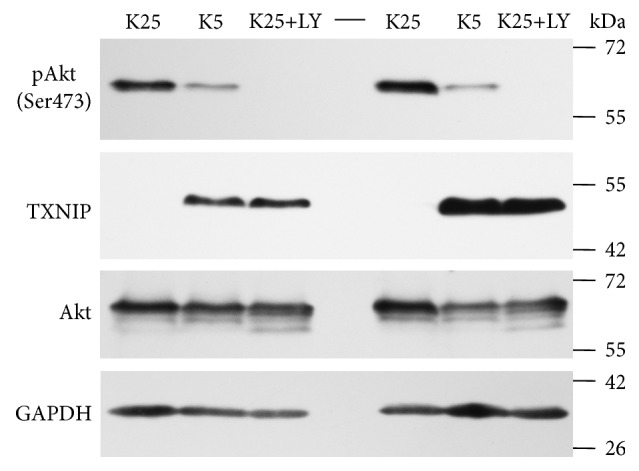
PI3K/Akt inhibition induces expression of TXNIP. Primary cultures of rat cerebellar granule neurons (CGN) were incubated with K25 as described in Materials and Methods. After 7 DIV some cells were treated with K5 during 3 h (negative control for Akt phosphorylation in Ser473 and a positive control for TXNIP expression), while other cells were treated with 30 *μ*M LY 294002 (inhibitor of PI3K/Akt pathway). Figure shows representative immunoblots from four independent experiments. GAPDH is the load control and Akt levels do not change. TXNIP: 50 kDa, Akt: 60 kDa, and GAPDH: 37 kDa.

Thus, we tested the effect of LY 294002 (5–30 *μ*M) on both pAkt levels and the expression of TXNIP. We found that LY 294002 (5–30 *μ*M) exerted a time and a concentration-dependent reduction in pAkt that closely correlated with a significant increase in TXNIP expression (Figure 10; Supplementary Figs.  5 and 6). These results strongly suggest that Akt regulates TXNIP expression similar to what is observed under K5 and Sts conditions. Additionally, these observations also suggest that PI3K regulates TXNIP expression through the regulation of Akt activity.

## 4. Discussion

It has been previously shown that ROS play a critical role in the apoptotic death of neurons. This has been extensively studied in cerebellar granule neurons [31, 32, 35, 36, 45, 46]; however, the mechanisms by which ROS modulate this process are still not clear. There are some evidences showing an involvement of ROS in the regulation of signaling pathways in apoptotic death, particularly the mitogen-activated protein kinases (MAPK) pathway. According to previous studies, ROS seem to mediate the activation of JNK and p38 pathways under apoptotic conditions in CGN [31, 32]. In addition, in nonneuronal cells, ROS induce the dissociation of thioredoxin 1 (Trx1) from ASK1, leading to its activation and the activation of JNK and p38 [47, 48]. Besides, the activation of Akt that inactivates ASK1 through the phosphorylation in Serine 83 is also redox sensitive [49]. Recent studies have also indicated a close relationship of TXNIP with several signaling proteins including Trx1, Trx2, and Akt [50–52]. It has been previously observed that TXNIP overexpression leads to apoptosis in cardiomyocytes [17] and the activation of the intrinsic mitochondrial pathway of apoptosis in INS-1 beta-cells [18].

Thus, it is interesting to understand the role of TXNIP in cell death of CGN and its relationship with ROS modulating signaling molecules such as Akt. In the present study, we found that two different apoptotic stimuli induce the expression of TXNIP in cultured cerebellar granule neurons (Figures 2 and 5). In the case of potassium deprivation (K5), a peak of TXNIP expression is observed after 2-3 h (Figure 2), which corresponds to the time of ROS generation by potassium deprivation (Figure 1(a)). Similar results were observed for staurosporine treatment that increased TXNIP expression by 3-4 h (Figure 5), which also correlates with the increase in the generation of ROS induced by staurosporine (Figure 1(b)).

It is interesting that the observed effect of both K5 and staurosporine was mediated by ROS as antioxidants markedly reduced the TXNIP expression induced by both K5 and Sts (Figures 3 and 6). Moreover, this effect was also abolished by NOX inhibitors, suggesting that ROS involved in this action are produced, at least partially, by a NOX (Figures 3 and 6). This is in accordance with previous observations showing that these two apoptotic conditions induce both the activation of NOX and ROS produced by a NOX in CGN [32, 53]. The involvement of ROS in the TXNIP expression induced by K5 and Sts was further supported by the observation that hydrogen peroxide was also able to induce markedly the expression of TXNIP (Figure 4).

It is interesting to mention that the TXNIP expression induced by K5 is higher than that observed by directly treating with hydrogen peroxide (Figure 4). This suggests that other conditions apart from ROS could be involved in the upregulation of TXNIP induced by K5. In that regard, it has been shown that the expression of TXNIP gene is modulated by the flow of calcium in CGN [54]. In that study a decrease in calcium levels, by blocking L-type voltage-gated calcium channels, led to an increase in the TXNIP mRNA expression. Conversely, a calcium ionophore evoked a reduction in TXNIP mRNA levels. Besides, the expression of TXNIP mRNA was not affected by pro- or antioxidant conditions. On the other hand, it is known that in CGN cultured in K25 calcium concentration is about 250 nM, while under 5 mM KCl (K5) calcium levels are by 50 nM [55]. We can therefore suggest that K5 condition is regulating TXNIP at both mRNA and protein levels by altering calcium and ROS levels, respectively, while hydrogen peroxide is upregulating TXNIP only at the protein levels. Therefore, these two conditions are probably contributing to inducing of different TXNIP levels under both K5 and H_2_O_2_ conditions.

TXNIP can participate in the cell death process through its interaction with the reduced form of thioredoxin by inhibiting its biochemical activity [11, 56]. In this regard, it has been proposed the concept of “Redoxisome” to refer to the complex Trx1-TXNIP as a signaling transducer relative to the redox state under normal and pathological conditions [51]. The TXNIP-Trx1 interaction has been extensively studied [6–10], including the possibility that the TXNIP-Trx1 interaction could play a critical role in neuronal death. Interestingly, Trx1 has been found to be a major binding partner of ASK1 [8, 9, 47]. Thus, TXNIP could regulate the Trx1-ASK1 interaction by binding to the catalytic cysteines of Trx1 and, therefore, inhibiting both Trx1 activity and its ability to bind to ASK1 [7, 48]. This condition would lead to the activation of the JNK/p38 pathway.

TXNIP could also induce cell death of CGN by Trx2 interaction. It has been reported in pancreatic beta-cells that TXNIP is localized primarily in the nucleus, but under oxidative stress it translocates to the mitochondria. In mitochondria, TXNIP binds to Trx2, thereby releasing ASK1 and resulting in ASK1 phosphorylation/activation and the initiation of the mitochondrial mediated apoptosis [50].

Recently, it was described the mechanism through which activation of PI3K/Akt signaling leads to inhibition of TXNIP protein and mRNA expression, in a model of synaptic activity [20, 57]. Papadia et al. [20] reported that TXNIP contains a functional FOXO site that mediates synaptic activity-dependent inactivation of the promoter. They propose that synaptic activity turns off TXNIP transcription by activating the PI3K/Akt pathway, which maintains FOXO phosphorylated and dissociated from the TXNIP promoter [20]. Thus, we propose here that the ROS generated by the apoptotic conditions, potassium deprivation and staurosporine, decrease Akt activation and therefore an increase in the expression of TXNIP.

To support this idea, we evaluated the activity of Akt and we found that in basal conditions (depolarizing conditions) Akt is fully activated in these neurons and both apoptotic conditions markedly suppressed the activation of Akt in a time-dependent manner (Figures 7 and 8). We noted temporal differences in the inhibition of this kinase; in the case of potassium deprivation (Figure 7), Akt was inhibited after one hour of treatment, while in the case of staurosporine this occurred after 30 min (Figure 8). This may be due to temporal differences related to the mechanisms involving both conditions. Furthermore, we found that Akt was sensitive to the redox state, since hydrogen peroxide was able to inactivate Akt (Figure 9).

In this regard, it was recently reported by Wani et al. [58] that ROS induced by PDGF cause the oxidation of the Cys124 of Akt2, modulating its inactivation. In addition, H_2_O_2_ induces the formation of disulfide bridges between Cys297 and Cys311 in Akt, as well as the subsequent dephosphorylation mediated by PP2A. Overexpression of Grx reduces Akt, which abolishes its binding to PP2A, then allowing a sustained Akt phosphorylation, inducing inhibition of apoptosis in cardiac cells [23], indicating an antiapoptotic action of Grx through a redox regulation of Akt. Another possibility is that the ROS generated by apoptotic conditions alter the redox state of PTEN, since it is known that H_2_O_2_ oxidizes Cys124 in the active site of PTEN that forms an intramolecular disulfide bond with Cys71 [59]. In addition, it was recently reported that ROS produced by NOX1 oxidize the Akt reactive cysteines promoting its interaction with PP2A, which inhibits Akt and therefore induces apoptosis in cardiomyocytes [60].

A possible Akt regulation on TXNIP expression in CGN was further evidenced by the observed increase in TXNIP expression by Akt inhibition (Figure 10; Supplementary Figs.  5 and 6). These results suggest that Akt regulates TXNIP expression similar to what is observed under K5 and Sts conditions. Additionally, our observations suggest that the Akt regulation on TXNIP expression involves the participation of PI3K.

Taken together, these results suggest that TXNIP could participate in the death of cerebellar granule neurons, probably through its interaction with Trx1 and/or Trx2, which would allow the activation of ASK1 and hence the activation of the signaling pathways downstream as JNK and p38. In these neurons, it has been shown that staurosporine and potassium deprivation cause apoptotic death by the activation of JNK and p38 pathways [31, 32]. On the other hand, Akt, besides the already known inhibition of ASK1, could also participate in the regulation of the expression of TXNIP. It is known that the inhibition of Akt induces an activation of p38 and JNK, whereas active Akt directly phosphorylates ASK1 Ser83, which leads to apoptosis inhibition [49]. Therefore, Akt could be acting at two different levels in the activation of apoptotic death.

We cannot discard the possibility that part of the mechanisms involved in CGN apoptotic death includes the Unfolded Protein Response (UPR) signaling. TXNIP regulates the Unfolded Protein Response (UPR) signaling and is considered a critical node in terminal UPR [61]. It is known that a reduction of TXNIP increases protein ubiquitination and splicing of the UPR regulated transcription factor Xbp1s [62]. TXNIP can also be induced by ER stress through the PERK and IRE1 pathways and represents a critical signal linking between ER stress and inflammation [63].

**Figure 11:**
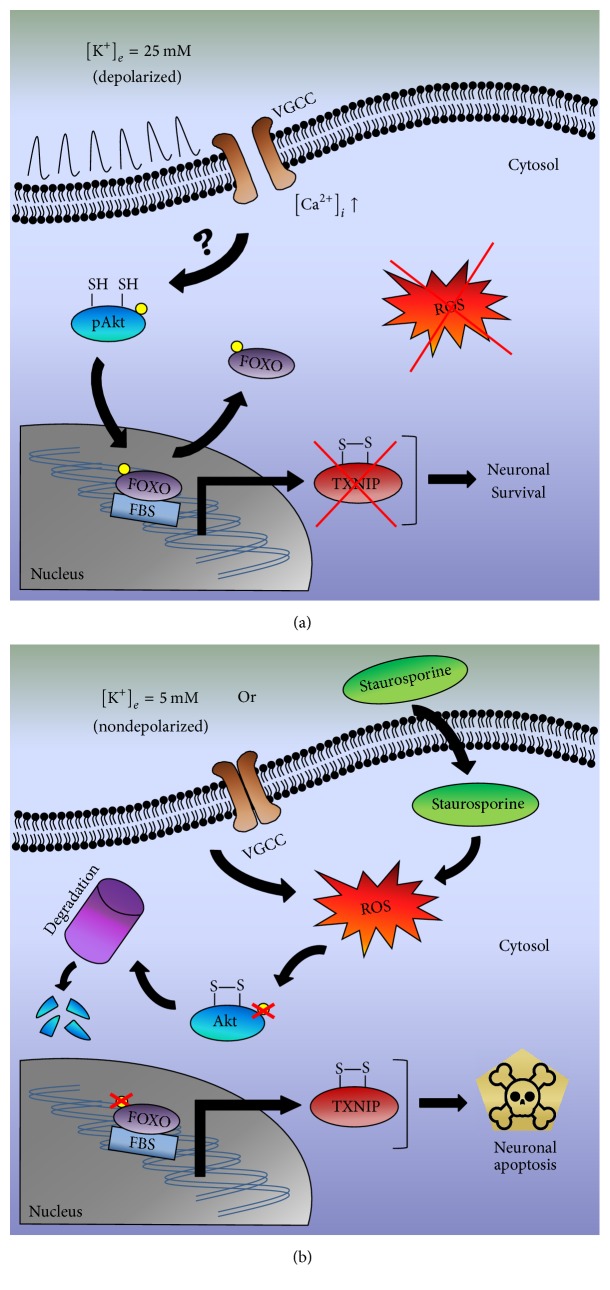
Schematic representation of the effect of apoptotic conditions on the expression of TXNIP in CGN. (a) In the model of cerebellar granule neurons, it has been shown that under depolarizing conditions in vitro (25 mM KCl, K25) the L-type voltage-gated Ca^2+^ channels are activated, allowing an increase of intracellular calcium. Under these conditions, our data indicate that there is no ROS generation and Akt is not active (phosphorylated in the Ser473). It is known that Akt can phosphorylate FOXO class of transcription factors, inhibiting its connection to FBS and promoting nuclear export of FOXO. This condition prevents the expression of the proapoptotic protein TXNIP, allowing the survival of the neuron. (b) In contrast, under potassium deprivation (K5) or staurosporine treatment, we observe a ROS generation, which may induce the inactivation of Akt and its subsequent proteasomal degradation. This situation could lead to the dephosphorylation of FOXO that, through its interaction with FBS, will induce the expression of TXNIP and the apoptotic process will proceed. VGCC, voltage-gated Ca^2+^ channels; FBS, FOXO binding site; pAkt, phosphorylated Akt; CGN: cerebellar granule neurons; FOXO, Forkhead box protein O.

In conclusion, our findings show that the inhibition of Akt induced by apoptotic conditions that generate ROS correlates with a TXNIP upregulation. In this context, the ROS generated by apoptotic conditions would allow the expression of TXNIP and likely contributes to the apoptotic death of cerebellar granule neurons. This study has shed new light on the mechanism involved in the death of the neurons in response to reactive oxygen species. This suggests that inhibition of TXNIP may represent a novel approach to reduce neuronal death induced by reactive oxygen species. A proposed scheme of the effects of apoptotic conditions (K5 and Sts) on Akt activation and TXNIP expression in CGN is presented in Figure 11.

## Supplementary Material

Supplementary Figure 1. ROS generation induced by K5 and Sts evaluated as DHEt positive cells. The generation of ROS in primary cultures of rat cerebellar granule neurons (CGN) was evaluated as DHEt positive cells. After 7 days in vitro (DIV) cells were transferred to K5 medium and DHEt positive cells were counted at different times, or were treated with Sts (0.5 μM) from 0.5 h to 5.5 h and DHEt positive cells were counted. Supplementary Figure 2. K5 decreases cell viability of cerebellar granule neurons. Cell viability was evaluated by measuring calcein (living cells) and IP (dead cells) staining, as well as the reduction of MTT. Cerebellar granule neurons (CGN) maintained in K25 for 7 DIV were transferred to a medium with K5 during 8 h, 24 h, 36 h and 48 h and viability was evaluated. Supplementary Figure 3. Sts decreases the cell viability of cerebellar granule neurons. Cell viability was evaluated by measuring calcein (living cells) and IP (dead cells) staining, as well as the reduction of MTT. Cerebellar granule neurons (CGN) maintained in K25 for 7 DIV were treated with Sts (0.5 µM) during 8 h, 24 h, 36 h and 48 h and viability was evaluated. Supplementary Figure 4. LY 294002 decreases cell viability of cerebellar granule neurons. Cell viability was evaluated by measuring calcein (living cells) and IP (dead cells) staining. Cerebellar granule neurons (CGN) maintained in K25 for 7 DIV were treated with three concentrations of LY 294002 for 24 hours and viability was evaluated. Supplementary Figure 5. TXNIP expression induced by LY 294002 is concentration-dependent. Primary cultures of rat cerebellar granule neurons (CGN) were cultured with K25. After 7 DIV some cells were treated with K5 during 3 h while other cells were treated with increasing concentrations of LY 294002 for 4 h and the levels of pAkt, Akt, TXNIP and GADPH were evaluated. Supplementary Figure 6. TXNIP expression induced by LY 294002 is time-dependent. Primary cultures of rat cerebellar granule neurons (CGN) were cultured with K25. After 7 DIV some cells were treated with 30 mM LY 294002 during the indicated times and the levels of pAkt, Akt, TXNIP and GADPH were evaluated.

## Abbreviations

CGN: Cerebellar granule neurons
K25: 25 mM potassium chloride
K5: 5 mM potassium chloride
Sts: Staurosporine
TXNIP: Thioredoxin-interacting protein
ROS: Reactive oxygen species.
